# dSir2 mediates the increased spontaneous physical activity in flies on calorie restriction

**DOI:** 10.18632/aging.100061

**Published:** 2009-06-22

**Authors:** Vijay Parashar, Blanka Rogina

**Affiliations:** Genetics and Developmental Biology, School of Medicine, University of Connecticut Health Center, Farmington, CT 06030-3301, USA; ^1^ Current Address: Department of Diagnostic Sciences, School of Dentistry, University of Detroit-Mercy, Detroit, MI 48208, USA

**Keywords:** dSir2, spontaneous physical activity, resveratrol, CR

## Abstract

Calorie restriction (CR) is the most effective way to increase life span and delay the onset of age-related symptoms in animals.  We have previously reported that CR affects a variety of physiological phenotypes in flies and results in dramatic behavioral, physical and demographic changes.  Here we show effects of low and high calorie levels on the spontaneous physical activity of flies. Wild type flies maintained on a low calorie diet exhibit higher spontaneous activity compared to flies on higher calorie diets. This increase is dependent on the presence of Sir2 since a low calorie diet does not increase the activity of dSir2 null flies. Similarly, increasing dSir2 activity by feeding flies resveratrol, a CR mimetic, increases spontaneous physical activity of flies on high caloric food. InDrosophila, spontaneous physical activity therefore closely mimics life span in its dependence on Sir2.

## Introduction

Aging in humans, and a wide variety of other animals, is characterized by the decline of physiological activity and function.  Learning, sensory function, reproduction, cardiac function and locomotor activity all senesce [1]. However, the restriction of dietary calories substantially slows the aging process and extends the lifespan of organisms including yeast, nematodes, fruit flies and rodents [2-6]. Beneficial effects of limited CR in primates and humans have been reported but the effects on maximal longevity are unknown [7,8].

Several lines of evidence suggest that the extension of life span due to CR in yeast, nematodes, and fruit flies is  mediated by an  increase in the activity of the  silent information regulator 2 (Sir2) gene [9-13].  Neither yeast, worms nor flies with reduced or absent Sir2 activity exhibit longer life span on reduced calorie media [12,14,15].  Similarly, survival of SirT1-null mice was decreased after exposure to CR [16]. Overexpression of *Sir2* genetically, or increase in Sir2 activity mediated by the drug resveratrol, increases life span without calorie restriction in yeast, nematodes, fruit flies and fish [13,17,18]. Consistent with this is the finding that overexpression of *SIRT1* in transgenic mice confers many of the physiological changes associated with CR [19]. In addition, as would be expected if the increase in life span due to CR is mediated by an increase in *dSir2* activity, CR does not increase the life span of  *dSir2* overexpressing flies [12,13]. However, there are several reports suggesting that in worms and yeast the Sir2 gene is not necessary for CR life span extension and that addition of resveratrol does not increase the longevity of yeast, worm or flies [20-26].

In addition to increased longevity, mice respond to CR with a general increase in physical activity that is also mediated by an increase in Sir2 activity [27]. Increases in five different measurements of physical activity such as walking, jumping and distance traveled, usually observed in CR mice, were not observed in mice lacking functional Sirt1, the mouse ortholog of yeast Sir2 [16,27]. In addition, transgenic mice over-expression *SIRT1* have increased rotarod performance [19]. Resveratrol given to mice on a standard diet mimics the effects of CR, reduces age-related pathology and improves performance on the rotarod with age [28]. Resveratrol given to mice on high calorie food also promotes numerous beneficial effects: increased insulin sensitivity, decreased organ pathology, increased activity of peroxisome proliferator-activated receptor-γ cooactivator 1α (PGC-1α) and AMP-activated protein kinase (MAMPK) and increased mitochondrial number [29,30]. In addition, this intervention also improves mouse neuromuscular function, and affects balance and motor coordination (improved rotarod performance, increased running time and consumption of oxygen in muscle fibers) [29,30].

*Drosophila* are typical of other animals, both in their pattern of senescence and in their response to CR. Flies decline with age by a variety of measures including learning, walking, flying, resting, phototaxis, jumping and general locomotor activity [31-33]. There are a number of reports studying locomotor activity of flies and its change with age. The reported studies used a variety of techniques, such as locomotion, geotaxis, fast phototaxis, RING assays, or Trikinetics activity monitors. Such techniques record the performance of the flies in particular events or continuously monitor spontaneous physical activity during 24 hours [33-39]. Several new sophisticated techniques for tracking of the 3D movements of flies have been described recently, designed for special uses such as behavioral analysis, free-flight response to motion and recording fly movements and gene expression [40,41]. Using Trikinetics activity monitors we found higher spontaneous physical activity of mixed population of flies under CR [42]. We used computer controlled "population activity monitors" to record spontaneous physical activity of each gender under several life extending conditions. Here, we report that CR is associated with increased spontaneous physical activity in Drosophila, similar to mammalian studies in mice, and that this increase is mediated by the fly Sir2 ortholog [16,27]. In addition, feeding the flies resveratrol, a CR mimetic, increased their spontaneous physical activity on a high calorie diet, further confirming the role of dSir2 in mediating increased spontaneous physical activity in CR flies.

## Results

### Daily spontaneous physical activity is affected by caloric intake

**Figure 1.**
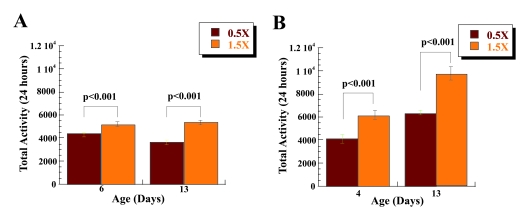
Low calorie diet is associated with increased spontaneous physical activity of *Drosophila*. Sum of 24-hour spontaneous physical activity of *Canton-S* male (**A**) and female (**B**) flies on a low (0.5X) and a high (1.5X) calorie food based on collected data for days 4 and 9. Both male and female flies on 0.5X food have increased spontaneous physical activity compared to the flies on l.5X food. The mobility was based on the mean mobility of 3 vials with 10 male or 10 female flies each, and expressed as mean total activity per vial during 24 hours +/- SEM. (**C**) Mean total 24 hours spontaneous activity of male and female *CS* flies on 0.5X and 1.5X food at age 4 and 9 expressed per vial. Statistical significance was determined by using two-tailed Student's t-test for independent samples.

We previously reported that the 24-hour activity of flies (mixed genders) depends on the calorie content of the food, with increased activity associated with a low calorie diet [47]. In order to determine gender-specific effects of diet on spontaneous physical activity, male and female Canton-S wild type flies were aged together on food with low (0.5X) and high (1.5X) calorie contents post-eclosion. The caloric content of 0.5X food is 50% that of the 1.0X food, and flies kept on 0.5X food have extended lifespan [42,43]. From 3 days of age, we recorded spontaneous activity of flies separated by gender in 3 groups of 10 male or female flies.  Each cohort was transferred into the population monitors with 0.5X or 1.5X food and placed in a temperature controlled incubator set at 25°C, with a 12 hour light-dark cycle. Using computer controlled population activity monitors, we were able to monitor spontaneous physical activity of the flies throughout most of their first 10 days of life. The days when the flies were passed to new vials were not used for calculations. There is a significant increase in the 24 hour spontaneous physical activity (total) observed in male flies kept at 0.5X food compared to flies kept on 1.5 food level at age 4 days [t(1, 58) = 7.21; η2= 0.47], Figure 1A, Table 1. A similar statistical difference in the total spontaneous activity of male flies kept on 0.5X and 1.5X was found at age 9, [t(1, 58) = 6.59; η2= 0.43], suggesting that the difference in activity is not only associated with very young age of 4 days. Female flies show a significant increases in spontaneous physical activity associated with low calorie food at age 4, [t(1, 58) = 9.15; η2= 0.59], but not at age 9, Figure 1B, Table 1. However, this result may be due to the high standard errors of the means observed in recorded mobility for female flies on 0.5X and 1.5X food levels at age 9. We also examined if there is a gender specific difference in the levels of physical activity of the flies in response to 0.5X vs. 1.5X food levels. There is no significant difference in the activity of the male and female flies on 0.5X food levels at both ages, nor on 1.5X food level at age 9. However, there is a statistically significant increase in the spontaneous physical activity of the female flies on 1.5X food levels at age 4 [t(1, 47.2) = -4.575; p<0.001; η2= 0.265]. Interestingly, the activity of the female flies is higher than males at both food levels at age 4, but lower at age 9 days, Figure 1C. These data further confirm that, like  mammals, male and female flies respond to a low calorie diet with increased spontaneous physical activity.

**Figure 2.**
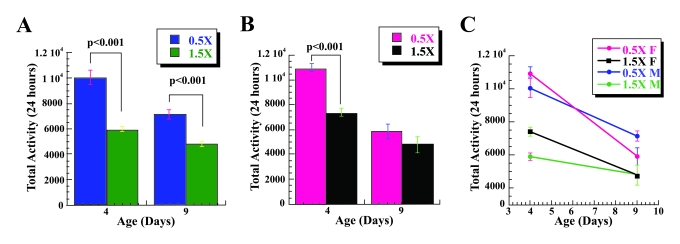
Increase in activity of male flies on low calorie food is mediated by *dSir2*. *dSir^4.5^*/*dSir^4.5^* (**A**) and *dSir^5.26^*/*dSir^5.26^* (**B**) homozygous *dSir2* null male flies have significantly lower 24-hour spontaneous physical activity on 0.5X (brown) & 1.5X (orange) calorie diet at two different ages. The mobility was based on the mean mobility of 3 vials with 10 male flies each, and expressed as mean total activity per vial during 24 hours +/- SEM. Statistical significance was determined by using two-tailed Student's t-test with unequal variances.

**Table 1.** Increased activity of flies on low calorie diet is mediated by dSir2.

Gender	Genotype	Food levels	Age	Mean Activity		SE	η^2^
						
M	*CS*	0.5	4	10006.00		547.12	0.47*
M	*CS*	1.5	4	5882.66	165.63		
M	*CS*	0.5	9	7112.33		287.12	0.43*
M	*CS*	1.5	9	4776.00		208.02	
						
F	*CS*	0.5	4	10893.66		266.94	0.59*
F	*CS*	1.5	4	7365.33		278.55	
F	*CS*	0.5	9	5876.33		551.43	0.17
F	*CS*	1.5	9	4759.33		594.62	
						
M	*dSir2^4.5^*/*dSir2^4.5^*	0.5	6	4279.33		77.64	0.502*
M	*dSir2^4.5^*/*dSir2^4.5^*	1.5	6	5195.00		91.22	
M	*dSir2^4.5^*/*dSir2^4.^*	0.5	13	3630.33		141.57	0.556*
M	*dSir2^4.5^*/*dSir2^46^*	1.5	13	5372.67		147.47	
						
M	*dSir2^5.26^*/*dSir2^5.26^*	0.5	4	4415.66		359.69	0.211*
M	*dSir2^5.26^*/*dSir2^5.26^*	1.5	4	6475.00		378.45	
M	*dSir2^5.26^*/*dSir2^5.26^*	0.5	13	6723.67		228.39	0.298*
M	*dSir2^5.26^*/*dSir2^5.26^*	1.5	13	10249.33		673.63	
						
M	*CS*	0.5 50Res	9	3224.67		41.65	0.222*
M	*CS*	0.5 100Res	9	3775.67		309.48	
M	*CS*	0.5 200Res	9	2358.33		156.99	
M	*CS*	0.5 EtOH	9	4645.50		229.85	
M	*CS*	1.5 50Res	9	5364.00		261.26	
M	*CS*	1.5 100Res	9	4663.67		170.89	
M	*CS*	1.5 200Res	9	5046.67		715.19	
M	*CS*	1.5 EtOH	9	3331.83		134.98	
						
M	*CS*	0.5 200Res	6	4192.67		136.62	0.23*
M	*CS*	0.5 EtOH	6	5116.33		248.51	
M	*CS*	1.5 200Res	6	7932.00		883.47	
M	*CS*	1.5 EtOH	6	4805.33		271.69	
						
M	*yw*	0.5 200Res	3	8880.67		611.66	0.248
M	*yw*	0.5 EtOH	3	7847.00		641.64	
M	*yw*	1.5 200Res	4	12721.67		259.56	0.724*
M	*yw*	1.5 EtOH	4	7028.00		381.95	

### Increased activity in flies on low calorie food is mediated by *dSir2*

Our life span studies reveal that the beneficial effects of CR on fly survivorship accrue and are mediated by *dSir2* [12]. In order to examine if increased activity under CR conditions is mediated by *dSir2*, we determined the activity of the *dSir^4.5^/dSir^4.5^* and *dSir2^5.26^/dSir2^5.26^*,*dSir2 *- homozygous null mutants on 0.5X and 1.5X food levels. Both *dSir^4.5^/dSir^4.5^* and *dSir2^5.26^/dSir2^5.26^* flies have lower spontaneous physical activity associated with CR, Figure 2A and B, Table 1. In contrast to wild type flies that have increased spontaneous physical activity on low calorie diet, we found that two different *dSir2* null homozygous flies have significantly lower spontaneous physical activity on 0.5X food compared to the 1.5X, Figure 2A and B, Table 1. Decreased activity on 0.5X food was observed at two different ages, 6 and 13 for *dSir^4.5^/dSir^4.5^* flies (age 6 [t(1, 58) = 7.64; p< 0.001; η2= 0.502], age 13 [t (1, 58) = 8.523; p< 0.001; η2= 0.556] and 4 and 13 for *dSir^5.26^/dSir^5.26^* flies (age 4 [t (1, 58) = 3.944; p< 0.001; η2= 0.211, age 13; [t (1, 35.6 = 4.957; p< 0.001; η2= 0.298]). While activity of *dSir^4.5^/dSir^4.5^* is similar in flies at ages 6 and 13, there is a significant increase in the spontaneous activity of *dSir^5.26^/dSir^5.26^* flies at age 13 compared to age 4 on 0.5X food [t (1, 49.1) = 5.417; p< 0.001; η2= 0.336] and 1.5X food [t (1, 45.648) = 4.885; p< 0.001; η2= 0.291]. Decreased physical activity observed in *dSir2* mutant flies on 0.5X food in comparison to 1.5 X food suggests that the presence of dSir2 is not only necessary for increased spontaneous physical activity of the flies on low calorie diet, but importantly, that dSir2 deficiency has negative effects on activity under CR conditions.

### Resveratrol restores normal physical activity to flies on a high calorie diet

**Figure 3.**
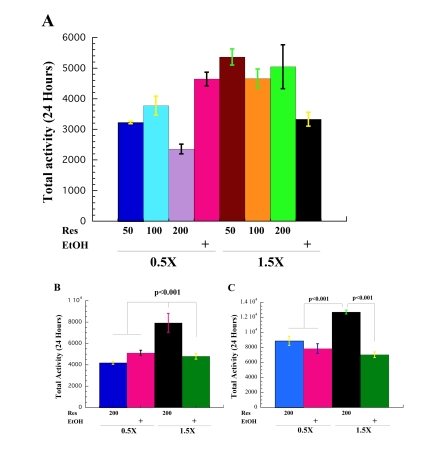
Resveratrol rescues low activity of the flies on high-calorie diet. (**A**) Effect of 50 μM, 100 μM and 200 μM of resveratrol in 0.5X (0.5X 50Res, 0.5X 100Res, 0.5 200Res) and 1.5X (1.5 50Res, 1.5 100Res, 1.5X 200Res) food on *CS* male spontaneous physical activity compared to activity of flies on 0.5X and 1.5X food that contain ethanol (0.5 EtOH and 1.5 EtOH) used as resveratrol solvent.  (**B**) Total daily spontaneous physical activity of male *CS* flies on 0.5X with 200 μM resveratrol (0.5 200Res) and 1.5X with μM 200 resveratrol (1.5 200Res) compared to the male flies on 0.5X and 1.5X food that contain ethanol (0.5 EtOH and 1.5 EtOH). The data are mean total 24s spontaneous activities collected independently for 3 vials with 10 flies each collected at age 9 (**A**) and 6 (**B**) days, except in A where there were 6 vials of 0.5X EtOH and 1.5X EtOH. A Tukey HSD post-hoc test was conducted on the food means to determine which means are pairwise statistical different from one another. Results of statistical analysis are in Supplementary Table 1A and Supplementary Table 1B. (**C**) Male *yw* wild type flies on 1.5X food with addition of 200 μM of resveratrol (1.5 200Res) have the highest activity compared to the flies on 1.5X EtOH, 0.5X 200Res and 0.5X EtOH. Flies were 3 (0.5X 200Res and 0.5X EtOH) and 4 (1.5XRes and 1.5EtOH) days old. Statistical significance was determined by using two-tailed Student's t-test with unequal variances.

The drug resveratrol, a polyphenolic STAC, increases the life span of yeast, worms, fruit flies and fish by activating Sir2 [13,17,18]. Moreover, this chemical activation of Sir2 increases the running time of mice fed a high fat diet [29,30]. We wanted to determine if dietary administration of resveratrol to flies maintained on a high calorie diet could boast the low spontaneous physical activity seen under these conditions. In order to evaluate the effects of different concentrations of resveratrol on fly spontaneous activity we recorded the spontaneous activity of male *CS* flies on 0.5X and 1.5X food with 50 μM, 100 μM and 200 μM of resveratrol or ethanol controls, Figure 3A. Statistical differences between means were found using a one-way analysis of variance (ANOVA) [F(7, 292)= 11.927, p<0.001, η2=0.222], Table 1. We found that addition of 50 μM, 100 μM or 200 μM of resveratrol increases the spontaneous physical activity of male flies on a high calorie diet to the levels of activity observed in control flies on 0.5X EtOH food, Figure 3A, Table 1. A similar increase in activity of flies on 1.5X Res food was observed at all three levels of resveratrol, suggesting that once the increase in physical activity reaches a certain threshold, additional increases in dSir2 activity do not further raise the physical activity of the flies. Addition of any of the three concentrations of resveratrol to the low calorie diet decreases the activity of flies compared to 0.5X EtOH controls, suggesting a negative effect of resveratrol on the spontaneous activity of CR flies, Figure 3A, Supplementary Table 1A and Supplementary Table 1B. However, the biggest negative impact on activity at 0.5X was observed with 200 μM of resveratrol. Statistical analysis is in Supplementary Table 1A and Supplementary Table 1B.

We also examined if addition of resveratrol increases the physical activity of younger flies fed a high calorie diet. We found that of 200 μM of resveratrol boasts the low spontaneous activity of male *CS* flies on 1.5X food to levels higher than 0.5X at age 6, Figure 3B, Table 1, Supplementary Table 1A and Supplementary Table 1B. An analysis of variance was performed to test mean differences of mobility between high calorie diet with addition of ethanol (1.5X EtOH) or resveratrol (1.5X Res), and low calorie diet with resveratrol (0.5X Res) or with ethanol (0.5X EtOH). A statistically significant difference was found between food groups for males at age 6 [F(3, 116) = 11.77; η2= 0.23). At age 6, spontaneous physical activity of flies on 1.5X Res was significantly increased compared to flies on all food conditions determined by Tukey's HSD post-hoc analysis, Supplementary Table 1A and Supplementary Table 1B. The flies on 0.5X with addition of 200 μM of resveratrol have the lowest activity. This suggests that the levels of dSir2 activity may directly determine fly activity- excess, as in case of addition of 200 μM resveratrol, or none as in case of *dSir2* mutant flies, decreases fly activity. Another explanation for low activity of *CS* flies on 0.5X Res food could be that concentration of 200 μM of resveratrol is too high and may have some negative effects on flies that are already under stress caused by CR.

In order to confirm that the addition of resveratrol increases the low spontaneous activity of flies on 1.5X food, we also determined the activity of *yw*, another wild type genetic background. As can be seen from the Figure 3C, addition of 200 μM resveratrol (1.5X 200Res) to high calorie diet significantly increases fly spontaneous physical activity compared all other food regimens: 1.5X with diluent ethanol (1.5X EtOH), or flies on 0.5X with resveratrol (0.5X 200Res) or diluent (0.5X EtOH). However, *yw* flies on 0.5X with resveratrol did not have the lowest activity as *CS* flies did. The different response of *yw *flies to addition of resveratrol to 0.5X food may be explained by different genetic backgrounds and slightly younger age. *yw* flies were 3 and 4 days old, while *CS* were 6 and 9 days old.

## Discussion

### Calorie restriction increases spontaneous physical activity in *Drosophila*

Caloric uptake affects many physiological functions [6,44]; this is especially true of spontaneous physical activity [45,46]. Within a range of caloric intake above starvation for flies, greater calorie consumption leads to higher body weight, and a higher rate of reproduction but a shorter life span [42]. We have previously reported that low calorie diet increases spontaneous physical activity of flies [42]. By using computer-controlled activity monitors, we were able to monitor spontaneous physical activity of flies longitudinally during the first 10 days of life. Comparisons of total spontaneous physical activity on days 4 and 9 of life reveal that total activity decreases with age but a CR-mediated increase in the activity of male flies is maintained. Females have increased activity associated with low calorie diet at age 4 but not at age 9.

### Sir2 mediates increased spontaneous physical activity of flies on low calorie food

Our studies on the molecular mechanisms underlying life span extension by CR suggest that *dSir2* mediates the CR response [11,12]. When Sir2 is overexpressed or activated by the drug resveratrol in yeast, worms, flies, or mice, there is an increase in life span on a rich diet that mimics the response to CR, even though nutrient supplies are superabundant [12,13,17,29,30,47]. Overexpression of SIRT1 in transgenic mice confers many of the same phenotypes as CR in mice, including increased performance  in a rotarod assay [19]. Consistent with Sir2-mediating the response to low calories, no further increases are obtained when Sir2 overexpression is combined with CR [12,13]. When Sir2 is reduced or absent, CR no longer induces longer life span in yeast, worms or flies [12,14,15]. Chen et al. (2005) reported that CR increases five different measurements of physical activity such as walking, jumping and distance traveled, but such increases were not observed in mice lacking the mouse orthologue of Sir2 [27]. Similarly, another group reported that SirT1-null mice don't increase their activity on CR and have lower total 24-hour activity on regular food [16]. Thus, we were prompted to examine whether increased spontaneous physical activity in *Drosophila* on low calorie diet is mediated by *dSir2*. We now report that increased spontaneous physical activity of *Drosophila* on low calorie food is mediated by dSir2, as is the case for mice. Furthermore, we found that the spontaneous physical activity of flies lacking Sir2 is lower on low calorie food compared to high. While Sir2 is necessary for increased mobility on low calorie, its absence actually has a negative effect on mobility when flies are raised on low calories. Several potential molecular mechanisms that could contribute to lower spontaneous physical activity of calorie restricted *dSir2* null flies come to mind. First, Sir2 has been implicated in energy metabolism and Sir2 deficiency, such as in SirT1-null mice, results in inefficient metabolism characterized by lower food utilization, altered mitochondria and metabolic rate and lower activity [16]. A role of Sir2 in regulating the amplitude of the circadian rhythm has also been described [48,49].

### Resveratrol restores spontaneous physical activity of flies on a high calorie diet

The drug resveratrol, a polyphenolic STAC, increases the life span of yeast, worms, fruit flies and fish by activating Sir2 [13,17,18]. Administration of resveratrol to wild type mice on either a regular diet or a high-calorie diet mimics effects of CR, postpones age-related pathology and has other benefits [28-30]. An effect of resveratrol on the physical activity in mice is also observed; it increases rotarod performance and endurance running, but decreases total spontaneous physical activity at high doses [29,30]. Similarly, addition of resveratrol postponed age-related decreases in locomotor activity of short-lived vertebrate fish, *N. furzeri* [18]. Consistently, *SIRT1* transgenic mice overexpressing *SirT1* have better performance on the rotarod [19]. We found that the administration of resveratrol to control flies on a high calorie diet increases their physical activity in two different control strains, *CS* and *yw*. Similar non-dose dependent restoration of activity on high calorie diet was observed when *CS* flies were subjected to three different doses of resveratrol suggesting the presence of a threshold for the increase in spontaneous activity of flies on high calorie diet that can be reached at certain levels of resveratrol, so that additional increases in resveratrol concentration and subsequent increases in dSir2 activity does not further increase spontaneous activity of the flies. Interestingly, addition of resveratrol to a low calorie diet decreases the spontaneous physical activity of *CS* flies but not *yw* flies. The decrease in spontaneous physical activity of calorie restricted *CS* flies was most pronounced in flies on the highest level of resveratrol of 200 μM. High levels of resveratrol may have some toxic effects, for instance negative effects were reported when rats and mice were exposed extremely high doses of resveratrol [28,50].

Similarly significant decrease in ambulatory locomotor activity and numbers of rears was observed in mice on high calorie diet with high doses of resveratrol, and in mice on high calorie diet after treatment with high doses of SRT1720, a potent SirT1 activator [51]. The effects of high levels of resveratrol may be through activation of Ser/Thr kinase AMPK, a known metabolic regulator that is also activated by CR or other targets and pathways known to be activated by resveratrol treatment [29]. The different response of *CS* and *yw* flies to the addition of resveratrol under CR conditions can be explained by different genetic backgrounds, which has been shown to effect survivorship, age-dependent changes in locomotor activity of male and female flies and response to CR [36,52].

Our results further indicate that the Sir2 orthologue, dSir2, mediates the CR-induced increase in spontaneous physical activity observed in flies. Consistent with this conclusion, the activation of Sir2 by resveratrol leads to an increase in activity on high calorie food. Interestingly, we found that the activity of flies on a low calorie diet is sensitive to the levels of dSir2 activity, too much or none results in lowered activities. Illustrating this is the fact that dSir2 null mutant flies on a low calorie diet have lower activity compared to the mutants on high calorie diet. Furthermore, the addition of resveratrol, a dSir2 activator, to 0.5X food similarly decreases *CS* flies activity. In this study, we used a computer assisted measure of spontaneous physical activity to extend our earlier findings on mobility in flies, and show that Sir2-mediated increases in spontaneous physical activity occur under CR and addition of resveratrol, conditions known to lead to Sir2-mediated increases in life span.

## Materials and methods

Fly stocks, food preparation and maintenance were described previously [12,45]. The *Canton-S* strain is the standard wild-type background line obtained from the Bloomington Stock Center. *dSir2^4.5^* and *dSir2^5.26^* mutant flies are null for *dSir2* gene (S. Smolik). Flies were maintained in a humidified temperature-controlled environmental chamber at 25°C (Percival Scientific) on a 12-hour light: dark cycle with light on at 6:AM.

Dietary calorie content of *Drosophila* food. Standard laboratory corn media as well as food marked as 0.5X and 1.5X were used. The two food levels are standardized as 1.0X being the food that has 100 g/L of sucrose (MP Biomedicals, Inc), 100 g/L of brewer's yeast (MP Biomedicals, Inc) and 20 g/L of agar [42,43].

Resveratrol (Sigma) dissolved in EtOH was added to the food during its preparation in final concentration of 50 μM, 100 μM and 200 μM. For control experiments the same volume of EtOH was added to the food. Food was prepared as previously described [13].

Spontaneous physical activity monitors. 20 male and 20 female flies were aged together on appropriate food since the day of their eclosion. On day 3 flies were separated by gender and three subgroups of 10 males or female flies were placed in population monitors and their physical activity was recorded every 10 minutes for the first 10 days of their life (*Drosophila* population monitor by Trikinetics Inc., Waltham, MA, USA). Reading chambers have circular rings of infrared beams at three different levels, which allow recording every time when fly crosses the rings. Activity monitors were kept in temperature control incubators set at 25°C on a 12-h light-dark cycle. The daylight period began at 6:00AM. Flies were replaced with a new set of flies of the same ages every two to three days. Recorded activities for the days when flies were replaced were not used for calculations.

Statistical analysis. A two-tailed Student's t-test was used for the analysis of the effects of 0.5X and 1.5X food levels on the mobility of wild type *CS* and*dSir2* null flies. Similar analysis was performed to analyze the effects of addition of 200 μM of resveratrol or EtOH to 0.5X and 1.5X food on the mobility of wild type *yw* male flies. A one-way analysis of variance (ANOVA) was performed to assess whether there are differences between mean activity of male *CS* flies on low (0.5X) and high (1.5X) calorie diet with addition of different doses of resveratrol or ethanol. A Tukey HSD post-hoc test was conducted on the mean mobility of wild type *CS* male flies to analyze the effects of addition of different doses of resveratrol or EtOH to 0.5X and 1.5X food on the mobility and to determine which means are pairwise statistically significantly different from one another.

## Supplementary table

Supplementary Table 1A Resveratrol rescues low activity of the flies on high calorie diet.

*The mean difference is significant at the 0.05 levels.

Supplementary Table 1-summarized The pairwise differences can be summarized as follows:

* = p < .05 ** = p < .01 *** = p < .001

 A Tukey HSD post-hoc test was conducted on the mean 24 hour spontaneous physical activity of male wild type CS flies kept on low food with 50 μM, 100?μM and 200 μM resveratrol (0.5 50Res, 0.5 100Res, 0.5 200Res), 0.5 low calorie food with ethanol, (0.5 EtOH) or high calorie food with 50 μM, 100 μM and 200 μM resveratrol (1.5 50Res, 1.5 100Res, 1.5 200Res) or ethanol (1.5 EtOH) to determine which means are paiwise statistically significantly different from one another. Flies were kept at 25°C during recording of the spontaneous physical activity. Flies were 9 days old.

Supplementary Table 1B Resveratrol rescues low activity of the flies on high calorie diet.

A Tukey HSD post-hoc test was conducted on the mean 24 hour spontaneous physical activity of male wild type CS flies kept on low food with 200 μM resveratrol (0.5 200Res), 0.5 low calorie food with ethanol, (0.5 EtOH) or high calorie food with 200 μM resveratrol (1.5 200Res) or ethanol (1.5 EtOH) to determine which means are paiwise statistically significantly different from one another. Flies were kept at 25°C C during recording of the spontaneous physical activity. Flies were and 6 days old.
