# Supplementary material for: dSir2 mediates the increased spontaneous physical activity in flies on calorie restriction
Source: Aging (Albany NY). 2009 Jun 22;1(6):529–41. doi: 10.18632/aging.100061 (PMC2806034; doi:10.18632/aging.100061)
Supplement: Supplementary Table 1A [file aging-01-529-s001A.doc]

**Supplementary Table 1: Resveratrol rescues low activity of the flies on high calorie diet**

A)

| Food 1 | Food 2 | Mean Difference  Food 1 –Food 2 | p-value |
| --- | --- | --- | --- |
|  |  |  |  |
| 0.5 50Res | 0.5 100Res | -551.0 | 0.924 |
|  | 0.5 200Res | 866.33 | 0.535 |
|  | 0.5 EtOH | -1420.83* | 0.008 |
|  | 1.5 50Res | -2139.33* |  |
|  | 1.5 100Res | -1438.00* | 0.033 |
|  | 1.5 200Res | -1822.00* | 0.002 |
|  | 1.5 EtOH | -107.16 | 1.000 |
|  |  |  |  |
| 0.5 100Res | 0.5 50Res | 551.00 | 0.924 |
|  | 0.5 200Res | 1417.33* | 0.038 |
|  | 0.5 EtOH | -869.83 | 0.336 |
|  | 1.5 50Res | -1588.33* | 0.011 |
|  | 1.5 100Res | -888.00 | 0.502 |
|  | 1.5 200Res | -1271.00 | 0.093 |
|  | 1.5 EtOH | 443.83 | 0.948 |
|  |  |  |  |
| 0.5 200Res | 0.5 50Res | -866.33 | 0.535 |
|  | 0.5 100Res | -1417.33* | 0.038 |
|  | 0.5 EtOH | -2287.16* | 0.000 |
|  | 1.5 50Res | -3005.66* | 0.000 |
|  | 1.5 100Res | 1-2305.33* | 0.000 |
|  | 1.5 200Res | -2688.33* | 0.000 |
|  | 1.5 EtOH | -973.50 | 0.200 |
|  |  |  |  |
|  |  |  |  |
| 0.5 EtOH | 0.5 50Res | 1420.83 | 0.008 |
|  | 0.5 100Res | 869.83 | 0.336 |
|  | 0.5 200Res | 2287.16* | 0.000 |
|  | 1.5 50Res | -718.50 | 0.591 |
|  | 1.5 100Res | -18.16 | 1.000 |
|  | 1.5 200Res | -401.16 | 0.970 |
|  | 1.5 EtOH | 1313.66 | 0.001 |
|  |  |  |  |
| 1.5 50Res | 0.5 50Res | 2139.33* | 0.000 |
|  | 0.5 100Res | 1588.33* | 0.011 |
|  | 0.5 200Res | 3005.66* | 0.000 |
|  | 0.5 EtOH | 718.50 | 0.591 |
|  | 1.5 100Res | 700.33 | 0.776 |
|  | 1.5 200Res | 317.33 | 0.997 |
|  | 1.5 EtOH | 2032.16* | 0.000 |
|  |  |  |  |
| 1.5 100Res | 0.5 50Res | 1439.00* | 0.033 |
|  | 0.5 100Res | 888.00 | 0.502 |
|  | 0.5 200Res | 2305.33* | 0.000 |
|  | 0.5 EtOH | 18.16 | 1.000 |
|  | 1.5 50Res | -700.00 | 0.776 |
|  | 1.5 200Res | -383.00 | 0.990 |
|  | 1.5 EtOH | 1331.83* | 0.016 |
|  |  |  |  |
|  |  |  |  |
| 1.5 200Res | 0.5 50Res | 1822.00* | 0.002 |
|  | 0.5 100Res | 1271.00* | 0.093 |
|  | 0.5 200Res | 2688.33* | 0.000 |
|  | 0.5 EtOH | 401.16 | 0.970 |
|  | 1.5 50Res | -317.33 | 0.997 |
|  | 1.5 100Res | 383.00 | 0.990 |
|  | 1.5 EtOH | 1714.83* | 0.000 |
|  |  |  |  |
| 1.5 EtOH | 0.5 50Res | 107.16 | 1.000 |
|  | 0.5 100Res | -443.83 | 0.948 |
|  | 0.5 200Res | 973.50 | 0.200 |
|  | 0.5 EtOH | -1313.66* | 0.001 |
|  | 1.5 50Res | -2032.16* | -0.000 |
|  | 1.5 100Res | -1331.83* | 0.016 |
|  | 1.5 200Res | 1.5 200Res | 0.000 |
|  |  |  |  |

*The mean difference is significant at the 0.05 levels.
