# Supplementary material for: dSir2 mediates the increased spontaneous physical activity in flies on calorie restriction
Source: Aging (Albany NY). 2009 Jun 22;1(6):529–41. doi: 10.18632/aging.100061 (PMC2806034; doi:10.18632/aging.100061)
Supplement: Supplementary Table 1-summarized — The pairwise differences can be summarized as follows: [file aging-01-529-s001-summarized.doc]

**Supplemental Table 1: Resveratrol rescues low activity of the flies on high calorie diet**

The pairwise differences can be summarized as follows:

|  | 0.5 50Res | 0.5 100Res | 0.5 200Res | 0.5 EtOH | 1.5 50Res | 1.5 100Res | 1.5 200Res | 1.5 EtOH |
| --- | --- | --- | --- | --- | --- | --- | --- | --- |
| 0.5 50Res | - | - | - | ** | *** | * | ** | - |
| 0.5 100Res |  | - | * | - | * | - | - | - |
| 0.5 200Res |  |  | - | *** | *** | *** | *** | - |
| 0.5 EtOH |  |  |  | - | - | - | - | ** |
| 1.5 50Res |  |  |  |  | - | - | - | *** |
| 1.5 100Res |  |  |  |  |  | - | - | * |
| 1.5 200Res |  |  |  |  |  |  | - | *** |
| 1.5 EtOH |  |  |  |  |  |  |  | - |

* = p < .05

** p < .01

*** = p < .001

A Tukey HSD post-hoc test was conducted on the mean 24 hour spontaneous physical activity of male wild type *CS* flies kept on low food with 50 μM, 100 μM and 200 μM resveratrol (0.5 50Res, 0.5 100Res, 0.5 200Res), 0.5 low calorie food with ethanol, (0.5 EtOH) or high calorie food with 50 μM, 100 μM and 200 μM resveratrol (1.5 50Res, 1.5 100Res, 1.5 200Res) or ethanol (1.5 EtOH) to determine which means are paiwise statistically significantly different from one another. Flies were kept at 25° C during recording of the spontaneous physical activity. Flies were 9 days old.
